# Effects of Yucca Extract on Nutrient Digestibility, Antioxidant Status, Estrus and Faecal Microorganism in Gilts

**DOI:** 10.3390/ani14233356

**Published:** 2024-11-21

**Authors:** Junjie Gao, Wenyan Wei, Chao Ji, Xujing Pan, Junlei Chang, Qianhou Zhang, Xilun Zhao, Xuemei Jiang, Ruinan Zhang, Lianqiang Che, Yan Lin, Zhengfeng Fang, Yong Zhuo, Bin Feng, Jian Li, Lun Hua, De Wu, Shengyu Xu

**Affiliations:** 1 Animal Disease-Resistance Nutrition, Ministry of Education, Ministry of Agriculture and Rural Affairs, Key Laboratory of Sichuan Province, Animal Nutrition Institute, Sichuan Agricultural University, Chengdu 611130, China; gaojunjie@stu.sicau.edu.cn (J.G.); 2022214007@stu.sicau.edu.cn (W.W.); chao.jien@outlook.com (C.J.); 2021214062@stu.sicau.edu.cn (X.P.); junlei@stu.sicau.edu.cn (J.C.); 71192@sicau.edu.cn (X.Z.); 71310@sicau.edu.cn (X.J.); 72022@sicau.edu.cn (R.Z.); che.lianqiang@sicau.edu.cn (L.C.); linyan@sicau.edu.cn (Y.L.); zfang@sicau.edu.cn (Z.F.); zhuoyong@sicau.edu.cn (Y.Z.); fengbin@sicau.edu.cn (B.F.); 14109@sicau.edu.cn (J.L.); hualun@sicau.edu.cn (L.H.); wude@sicau.edu.cn (D.W.); 2 Shandong Huachang Animal Health Products Co., Ltd., Jinan 250000, China; ytzhzqh@163.com

**Keywords:** yucca extract, nutrient digestibility, antioxidant status, faecal microorganism

## Abstract

The objective of this study was to investigate the effects of yucca extract (YE) supplementation on estrus, nutrient digestibility, antioxidant capacity and fecal microorganisms of gilts. Twenty gilts were randomly divided into two groups: basal diet (CONT) and basal diet + 0.25 g/kg YE (YETG). The results showed that supplementing 0.25 g/kg YE in the diet of gilts significantly increased the apparent digestibility of dietary energy, crude fat and crude protein (*p* < 0.05). In addition, YE could also improve the antioxidant capacity of gilts, significantly increase the serum total antioxidant capacity (T-AOC) activity and decrease the malondialdehyde (MDA) content of gilts (*p* < 0.05). In terms of fecal microorganisms, YE significantly increased the Shannon index and Simpson index of fecal microorganisms of gilts (*p* < 0.05), decreased the abundance of *Proteobacteria*, *Actinobacteriota* and *Streptococcus* sp., and increased the abundance of *Muribaculaceae* and *Prevotalla* sp. in the feces of gilts (*p* < 0.05). In conclusion, dietary YE increased the apparent digestibility of nutrients, improved the antioxidant status of gilts and increased the α diversity of fecal microorganisms. These results provide a reference for the application of YE in gilts production.

## Simple Summary

Yucca extract (YE) is natural additive derived from the Yucca genus of plants, which is rich in active substances such as saponins, polyphenols, and polysaccharides. As a safe new type of feed additive, YE can play multiple biological roles in animal production, including promoting growth, improving reproductive performance, enhancing feed utilization efficiency, and improving animal husbandry. Our results showed that supplementing the diets of gilts with 0.25 g/kg of YE can enhance apparent nutrient digestibility, improve antioxidant status and increase fecal microbial diversity, ultimately benefiting the overall health status of gilts. The aim of this study is to provide experimental evidence for the application of yucca extract in animal production.

## 1. Introduction

Animals in the process of breeding may encounter various diseases, stress responses, and growth disorders. These issues not only impact animal welfare but also increase breeding costs and reduce economic benefits. Plant extracts have been widely utilized as a safe and green feed additive in both animal husbandry and food production [1,2]. YE is a liquid or powder substance extracted from the roots, leaves, flowers and bark of yucca plants. Its main active components include saponins, polyphenols and polysaccharides, which have antioxidant, anti-inflammatory, antiviral and other effects [3]. The United States Food and Drug Administration (FDA) classifies it as Generally Recognized as Safe (GRAS) and has been widely used in food additives, pharmaceuticals, agricultural products and animal feed [4]. As a new feed additive, YE not only has good effects in promoting growth, regulating the immune system and reducing breeding costs [5,6,7] but also demonstrates the capability to decrease ammonia concentrations within livestock barns, thereby enhancing the overall breeding environment [7,8].

Estrus signifies the sexual maturity and reproductive capability of gilt. The earlier the first estrus occurs in gilts, the more mature their reproductive organs and bodily development are, allowing them to enter the reproductive cycle sooner and enhancing their utilization rate and reproductive efficiency [9]. The management of gilt-first estrus is crucial to their reproductive performance and economic benefits. Research has indicated that high concentrations of ammonia gas can impair the olfactory function of the noses of gilts, resulting in a delay in their pubertal onset [10]. Furthermore, the active component saponin in YE has the effect of binding with ammonia to improve nitrogen metabolism in the organism, thereby reducing ammonia emissions [11,12]. This indicates that the rational use of yucca extract could provide new insights and approaches for improving the pig barn environment and optimizing the first estrus stage of gilts.

The gut microorganism plays a critical role in the host body by breaking down complex carbohydrates, proteins and other nutrients, producing metabolites such as short-chain fatty acids (SCFAs), providing energy to the host, and promoting the growth and repair of intestinal cells [13]. Therefore, the health status of intestinal microorganisms directly affects the absorption and utilization efficiency of nutrients in gilts. Studies have found that YE can regulate the apoptosis of intestinal mucosal cells, protect the integrity of intestinal mucosal tissue, increase the height of intestinal villi, enhance the intestinal barrier, and improve the efficiency of nutrient absorption [14,15]. Sun et al. [15] found that adding YE increased the β-diversity of gut microbes and increased the number of beneficial bacteria, such as *Lactococcus* sp. and *Bacteroides* sp. In addition, YE can improve the apparent digestibility of dietary nutrients. A study conducted by Fan et al. [16] found that dietary YE can improve the activities of amylase, lipase, trypsin and alkaline phosphatase in the jejunum of weaned piglets, and accelerate the decomposition and absorption rate of nutrients. In addition, in the poultry study, it was found that YE can significantly improve the digestibility of crude protein, crude fat and crude fiber in the feed of ileum, so as to improve the feed utilization rate of broilers and increase the average daily gain [17,18]. Therefore, the purpose of this study was to investigate the effects of dietary YE on estrus, apparent digestibility of feed, antioxidant capacity and fecal microorganisms of gilts.

## 2. Materials and Methods

The experiment was conducted in the teaching and research base of the Animal Nutrition Research Institute of Sichuan Agricultural University and this experiment was approved by the Animal Care and Use Committee of Sichuan Agricultural University (ethical approval code: SICAU20211209).

### 2.1. Animals and Experiment Design

Twenty healthy Large White × Yorkshire (LY) gilts, all with a similar body weight of 105 ± 15 kg, were randomly assigned to one of two treatments: basal diet (CONT) and basal diet + 0.25 g/kg YE (YE). YE was gifted by Shandong Huachang Animal Health Products Co., Ltd. (Jinan, China). The main components of YE included yucca saponins, yucca polysaccharides, and yucca polyphenols. The guaranteed value of this product was yucca saponin content ≥12%. During the feed preparation process, YE was first evenly mixed with vitamins, minerals, and carriers, and then blended with bulk ingredients such as corn and soybean meal. The experimental phase lasted for 35 days, starting from the day when the gilts reached a body weight of approximately 105 kg and concluding on the 35th day. The basic diet is shown in Table 1, and diets were formulated according to NRC 2012 to meet or exceed the nutritional requirements of animals. The ambient temperature in the piggery was controlled between 18 and 23 °C. Gilts were fed twice a day at 8:00 a.m. and 15:00 p.m. Each pig was fed 2.8 kg of feed per day. In the whole experiment procedure, gilts were allowed to drink water freely.

### 2.2. Sample Collection

During each feeding process, feed samples were collected randomly, and the nutritional composition of the diet was determined after the samples were crushed according to the standard [19].

The experiment period was 35 days. On the last day of the experiment, seven gilts per group were randomly selected for blood samples. A 10 mL blood was collected into centrifuge tubes from the anterior vena cava. The serum samples were obtained by centrifuging blood samples at 3500× *g* for 15 min, whereafter they were stored at −20 °C for the analysis.

On the 33rd day of the experiment, fresh feces without pollution were collected for 3 consecutive days, and the digestion experiment was carried out by the acid-insoluble ash (AIA) method: add 10% sulfuric acid 10 mL (Chengdu, China) per 100 g of fresh feces, drop 2 drops of toluene (Chengdu, China), mix well, and store in the refrigerator at −20 °C. Following the completion of the experiment, the collected fecal samples were baked at 60–65 °C to constant weight and then crushed through a 40-mesh screen and placed in a −20 °C refrigerator to be measured for the determination of the apparent digestibility of feed.

On the last day of the experiment, 7 gilts were randomly selected from each treatment group, and fecal samples were collected using the rectal stimulation method. The freshly collected and uncontaminated feces were placed into 1.5 mL frozen tubes and then stored in a −80 °C freezer for fecal microbial analysis.

### 2.3. Determination Index and Method

During the experimental period, the gilts were observed daily. If signs such as swelling of the vulva, reddening of the vulvar color, secretion of mucus from the vulva, and the presence of standing reflex were observed, the gilt was deemed to be in estrus, and the estrus time was recorded. Gilts were weighed and backfat thickness was measured on an empty stomach on day 1 and at the end of the experiment. We calculated the average daily gain [ADG, ADG = (Final body weight − Initial body weight)/days of feeding] of the gilt. The backfat thickness was measured at a point that is 6.5 cm away from the dorsal midline, directly upward along the tangent line of the last rib on the gilt. Based on the daily energy requirements of the gilt, each gilt was fed 2.8 kg of feed per day.

Acid-insoluble ash (AIA) was used as an endogenous indicator to determine the apparent digestibility of feed energy, crude protein, and crude fat content. The formula for apparent nutrient digestibility is apparent nutrient digestibility (%) = [1 − (AIA content in the diet/AIA content in the fecal sample) × (nutrient content in the fecal sample/nutrient content in the diet)] × 100.

Total antioxidant capability (T-AOC), the activities of superoxide dismutase (SOD), total glutathione (T-GSH) and malonaldehyde (MDA) in serum were estimated using commercial kits (Nanjing Jiancheng Bioengineering Institute, Nanjing, China), and the measurement procedures were carried out according to the instructions.

The fecal samples of gilts were sent to Beijing Novogene Co., Ltd. (Beijing, China) for 16S rRNA sequencing. The general steps involved in the process were as follows: Firstly, genomic DNA was extracted using the Magnetic Soil and Stool DNA Kit (TianGen, Beijing, China; Catalog #: DP712). Subsequently, the DNA concentration was assessed via 1% agarose gel electrophoresis. Then, the extracted DNA was amplified by PCR and further analyzed by electrophoresis using 2% agarose gel. After PCR amplification, the products were mixed in equidensity ratios. Subsequently, the mixed PCR products were purified using the Universal DNA Purification Kit (TianGen, Beijing, China; Catalog #: DP214). Following purification, the qualified PCR products were pooled and recovered. Finally, the NEB Next Ultra™ II FS DNA PCR-Free Library Prep Kit (New England Biolabs (Ipswich, MA, USA)) was utilized for library preparation. The prepared library was then quantified using Qubit and qPCR. Once the library was deemed qualified, it was sequenced on the NovaSeq 6000 platform with PE 250 sequencing. Subsequently, we conducted in-depth analysis of sequencing results through ASV clustering analysis, α-diversity analysis, β-diversity analysis, and species difference analysis, aiming to comprehensively explore the composition characteristics and community structure of microorganisms.

### 2.4. Statistical Analysis

SPSS27.0 software was used to conduct an independent sample *t*-test, all data were tested for normality and homogeneity of variance, and statistical analysis was conducted. The index measured was taken as the statistical unit, and the results were expressed as “mean ± standard error”, where *p* < 0.01 meant that the difference was extremely significant, and *p* < 0.05 meant that the difference was significant. 0.05 ≤ *p* < 0.1 indicates a trend.

## 3. Results

### 3.1. Effects of YE on Estrus and Growth Performance of Gilts

Compared with the CONT group, YE had no significant effect on the estrus date of gilts (Table 2). Similarly, YE had no significant effect on the body weight of gilts on the day of estrus.

YE had no significant effect on ADG, end weight and backfat deposition in gilts compared with the CONT (Table 2).

### 3.2. Effects of YE on Apparent Digestibility of Nutrients of Gilts

Compared with the CONT group, YE treatment significantly improved the apparent digestibility of dietary energy, dry matter, crude fat and crude protein (*p* < 0.05, Table 3).

### 3.3. Effects of YE on Serum Antioxidant Capacity of Gilts

Compared with the CONT group, YE treatment significantly increased serum T-AOC content and decreased serum MDA content of gilts (*p* < 0.05, Table 4). There was no significant effect on other antioxidant indexes.

### 3.4. Effects of YE on Fecal Microorganisms of Gilts

#### 3.4.1. Sequencing Data and ASV Clustering Analysis

**Figure 1:**
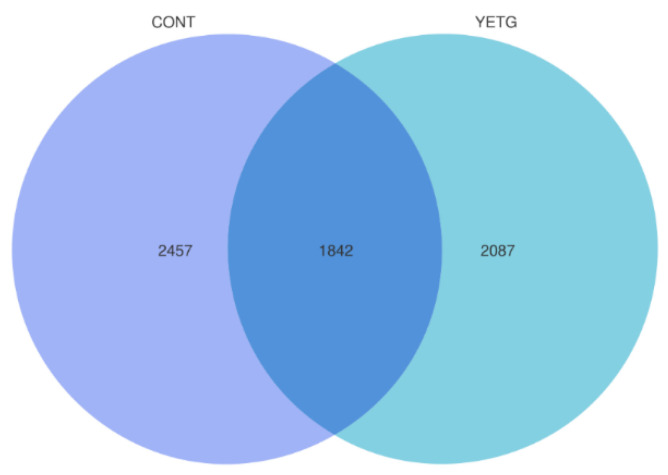
Effects of YE on the number of fecal microbial genes of gilts.

The number of ASVs shared by both the CONT and YETG groups is 1842 (Figure 1), with 2457 ASVs unique to the CONT group and 2087 ASVs unique to the YETG group.

#### 3.4.2. Sample Complexity Analysis

**Figure 2:**
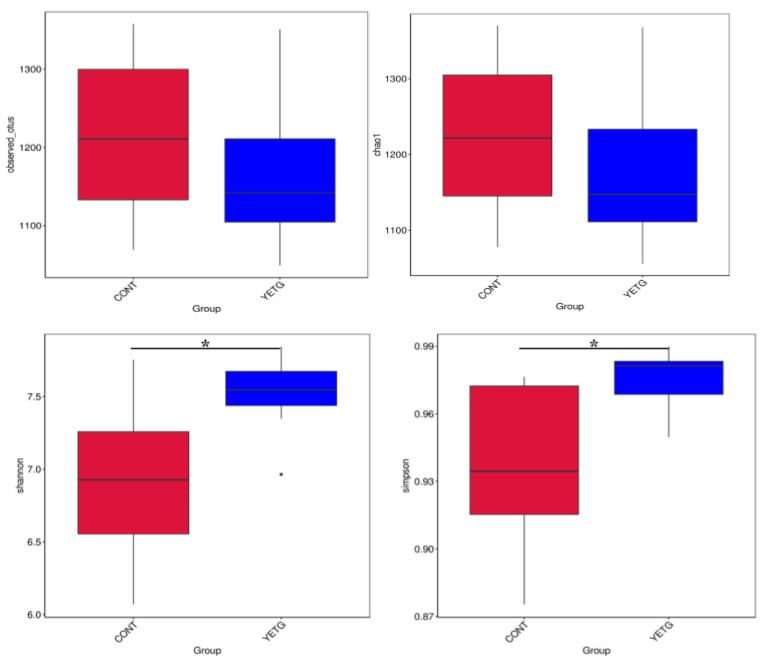
Effects of YE on fecal microbial α diversity of gilts. *, *p* < 0.05.

The results of specific effects on fecal microbial diversity are shown in Figure 2. The observed species, Chao1, Shannon, and Simpson indexes indicate that dietary supplementation of YE significantly affected the α diversity of fecal microflora. In terms of α diversity, compared with the CONT group, YE significantly increased the diversity of the Shannon index and Simpson index (*p* < 0.05, Table 5). There was no significant effect on the observed species and Chao 1 index.

**Figure 3:**
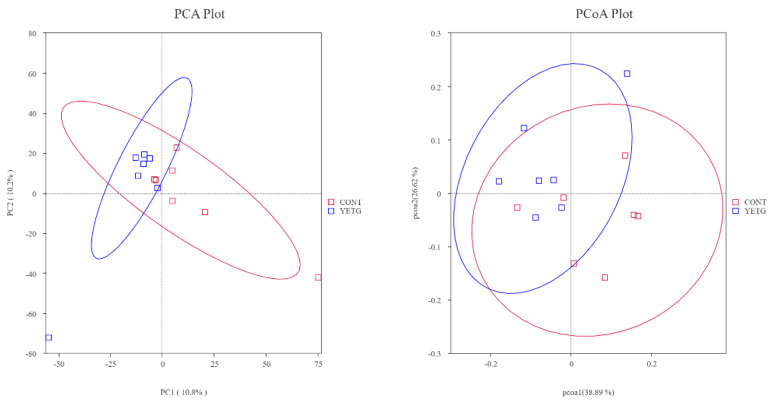
Effects of YE on fecal microbial β diversity of gilts.

As shown in Figure 3, PCA analysis based on ASV’s physical distance and PCoA analysis based on unweighted_unifrac distance found that dietary YE supplementation had no significant difference in fecal β diversity of gilts.

#### 3.4.3. Relative Abundance Analysis of Species

**Figure 4:**
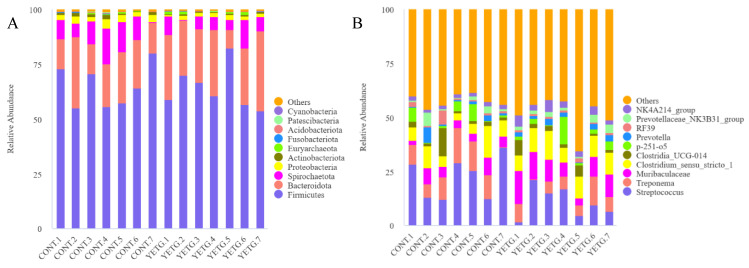
Effects of YE on fecal microbiota composition of gilts. Relative abundance at the phylum level of gilts (**A**). Relative abundance at the genus level of gilts (**B**). CONT, the basal diet; YETG, the basal diet + 0.25 g/kg yucca extract.

Figure 4 shows the relative abundance of microorganisms at the phylum level and genus level in the feces of gilts supplemented with YE. Firmicutes and Bacteroidetes were the main microorganism groups in gilts at the phylum level. Dietary YE supplementation significantly reduced the relative abundance of Proteobacteria and Actinobacteriota at the phylum level (*p* < 0.05, Table 6), but had no significant effect on the abundance of other top 10 species. At the genus level, dietary addition of YE significantly decreased the relative abundance of *Streptococcus* sp. and increased the relative abundance of *Muribaculaceae*, *Prevotella* sp., *RF39* and *NK4A214_group* (*p* < 0.05, Table 7). But there was no significant effect on the abundance of other microbes in the top 10 in species abundance.

#### 3.4.4. LEfSe Analysis of Different Species Between Groups

**Figure 5:**
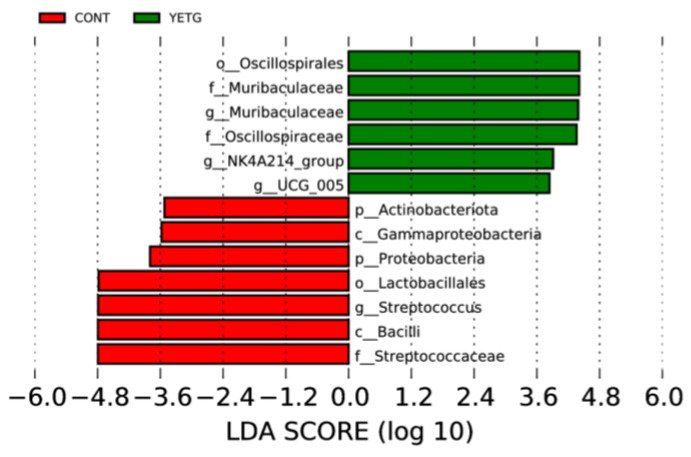
Distribution map of LDA values of different species.

As can be seen from Figure 5, there were significantly different microorganisms in CONT and YETG. At the phylum level, species with significant differences included Actinobacteriota and Proteobacteria. On the class level, Gammaproteobacteria and Bacilli showed significant differences. The species with significant differences at the family level include Muribaculaceae, Oscillospiraceae, and Streptococcaceae. Species that differ significantly at the genus level include Muribaculaceae, NK4A214_group, UGG_005 and Streptococcus.

## 4. Discussion

YE contains rich active ingredients, which can be used as feed additives to improve animal performance. The research conducted by Fan et al. [16] demonstrated that the addition of YE to the diet significantly increased ADG and ADFI and decreased the feed-to-gain ratio (F/G) of weaned piglets [20]. However, we found that YE did not have a significant effect on the growth performance of gilts, possibly because the growth of gilts tended to be stable in the later period. Additionally, in this study, the addition of YE did not affect the estrus time of gilts, which may be related to the fact that the dosage and duration of YE administration were not suitable to elicit a significant response during estrus. Whether YE can improve the estrus condition of sows by reducing ammonia emissions warrants further research.

Research has shown [21] that the addition of YE can promote the digestion and absorption of feed nutrients in animals. Our study also found the effects of YE on the apparent digestibility of nutrients. Dietary YE supplementation can significantly improve the apparent digestibility of energy, crude protein and crude fat. Consistent with previous studies by Min et al. [22], dietary supplementation of YE significantly increased the digestibility of dry matter, crude ash and crude protein. Similarly, adding YE to the diets of weaned piglets can significantly improve the nutrient digestibility of weaned piglets [16]. When 120 mg/kg YE was added to the growing pig diets, the apparent digestibility of dry matter, crude protein and essential amino acids in the diet was significantly increased [23]. The beneficial effects of YE on nutrient utilization may be attributed to its main active ingredient, saponins, which promote fat emulsification due to its surface activity and ability to delay the passage of intestinal chyme [24].

Oxidative stress is one of the important factors that inhibit the growth of animals, which can cause cell damage and induce disease [25]. It has been found that YE can effectively alleviate the oxidative stress state of animals [26,27,28]. In the study of weaned piglets, it was found that dietary YE supplementation can improve the antioxidant status of weaned piglets, significantly increase the plasma T-AOC and catalase (CAT) contents, and reduce the MDA content of weaned piglets [20]. In addition, in the study of pregnant sows, it was found that YE can significantly increase the serum CAT activity of sows, reduce MDA content, and improve the antioxidant capacity of sows [12]. Kucukkurt et al. [28] reported that 100 or 200 mg/kg of YE can significantly reduce the concentration of MDA in the blood of rats. Similar to this study, dietary supplementation with YE can significantly increase serum T-AOC activity and decrease MDA levels, thereby enhancing the antioxidant capacity of gilts. These results are consistent with previous studies. The antioxidant function of YE is primarily attributed to the phenolic hydroxyl groups in its saponins [29], which serve as hydrogen donors to oxygen free radicals generated during lipid peroxidation, thereby inhibiting the formation of hydroxyl peroxides and safeguarding against oxidative damage [26,30]. Furthermore, studies have also found that saponins can improve antioxidant levels by regulating the Nrf2 signaling pathway [11]. The mechanism by which YE exerts its antioxidant effects still awaits further research.

Intestinal microbiota plays an important role in maintaining host health [31] and is closely related to body health [32]. In this study, dietary YE supplementation significantly increased the Shannon index and Simpson index of fecal microbial α diversity of gilts and increased the abundance and diversity of the fecal microbial community. Some studies have found that dietary supplementation of YE can significantly increase the cecal microbial Chao1 index and cecal α diversity of weaned piglets [20]. Furthermore, Sun et al. [6] found that YE could increase the β-diversity of intestinal microorganisms and the number of beneficial bacteria. However, in the present study, YE did not have a significant impact on the β-diversity of feces in gilts. Therefore, supplementation of YE in the diet can improve the diversity and abundance of animal intestinal microorganisms.

Intestinal microorganisms can reflect the metabolic state of the body and the body’s health to a certain extent [33]. Similar to previous studies, the dominant bacterial phylum in sow feces are Firmicutes and Bacteroidetes [34,35,36]. Dietary YE supplementation had no significant effect on Firmicutes and Bacteroidetes abundance. However, the relative abundances of Proteobacteria and Actinobacteriota were significantly decreased by dietary YE supplementation. Proteobacteria contains a variety of pathogenic bacteria [37]. A reduction in their relative abundance may mean a reduction in the number of these potentially pathogenic bacteria in fecal, thereby reducing the risk of disease infection in gilts and contributing positively to their overall health. Actinobacteriota is involved in the synthesis of secondary metabolites such as antibiotics and toxins, suggesting that the addition of YE to the diet reduces the synthesis of these secondary metabolites, which is beneficial to intestinal health. At the genus level, dietary YE supplementation significantly increased the relative abundance of *Prevotella*. Some studies have found that *Prevotella* is crucial to lipid, carbohydrate, fibrous material and protein metabolism [38,39,40]. In this experiment, YE improved the apparent digestibility of dietary energy, crude protein and crude fat of gilts, which may be attributed to the relative abundance of *Prevotella*. At the same time, *Prevotella* can also improve intestinal immunity and have anti-inflammatory properties [41,42]. These results indicate that dietary YE supplementation can improve intestinal health. Some studies have found that certain Streptococcus has the function of metabolizing fructose [43], but Streptococcus is related to the occurrence of various metabolic disorders [44]. In this study, dietary YE supplementation significantly decreased the relative abundance of Streptococcus at the genus level. *Muribaculaceae* exhibit salutary impacts on intestinal dysbiosis by virtue of their immunomodulatory capabilities and regulation of gut homeostasis, thereby contributing to a healthier intestinal environment [45,46]. In addition, *Muribaculaceae* also participated in the degradation of complex carbohydrates [47,48], and dietary YE supplementation significantly increased the relative abundance of fecal *Muribaculaceae*. These results indicate that dietary YE may increase the abundance of beneficial bacteria, increase the abundance of microorganisms involved in digestion, and thus increase the digestibility of nutrients in the diet. At the same time, it can inhibit the growth of potential pathogenic bacteria, thereby improving the intestinal health of gilts.

## 5. Conclusions

In summary, the findings of this study indicate that supplementing the diets of gilts with 0.25 g/kg of YE can increase the apparent digestibility of dietary, improve the antioxidant status and enhance fecal microbiota α-diversity. This experiment provides practical guidance for the application of YE in the diets of gilts to improve the nutrient apparent digestibility of nutrients and antioxidant status. Further research is needed to determine whether the impact of YE on the antioxidant status of gilts will affect their reproductive performance.

## Figures and Tables

**Table 1:** Composition and nutrient contents of basal diets (as-fed basis, %).

Ingredients	Content
Corn	84.81
Soybean meal	10.00
Fish meal	1.80
Soybean oil	0.50
Limestone	0.80
Dicalcium phosphate	0.80
NaCl	0.30
L-Lys-HCI (78.5%)	0.41
DL-Met (99%)	0.05
L-Thr (98%)	0.13
L-Trp (98%)	0.03
Choline chloride (50%)	0.12
Vitamin premix ^a^	0.05
Mineral premix ^b^	0.20
Total	100.00
Nutrient composition ^c^	
Total energy (MJ/kg)	14.04
Crude protein, %	12.53
Crude fiber, %	2.55
Calcium, %	0.66
Total phosphorus, %	0.49
Available phosphorus, %	0.26
Total Lys, %	0.88
Total Met, %	0.28

^a^ The vitamin premix provided the following per kilogram of complete diets: vitamin A, 12,000 IU; vitamin D3, 2400 IU; vitamin E, 100 IU; vitamin K3, 4.8 mg; vitamin B1, 2 mg; vitamin B2, 7.2 mg; vitamin B6, 3.6 mg; vitamin B12, 25 μg; pantothenic acid, 25 mg; biotin, 0.48 mg; folic acid, 4 mg; nicotinic acid, 40 mg. ^b^ The mineral premix provided the following per kilogram of complete diets: Fe, 120 mg; Cu, 12 mg; Mn, 25 mg; Zn, 70 mg; I, 0.3 mg; Se, 0.35 mg. ^c^ The nutrient levels of crude protein, crude fiber, calcium and total phosphorus were calculated values.

**Table 2:** Effects of YE on estrus and growth performance of gilts.

Items	CONT	YE	*p*-Values
Estrus (day)	21.80 ± 0.76	20.70 ± 0.54	0.252
Weight on estrus day (kg)	119.45 ± 3.16	123.19 ± 2.29	0.357
Initial weight (kg)	104.94 ± 3.18	107.57 ± 2.83	0.548
End weight (kg)	128.34 ± 2.57	133.53 ± 1.91	0.131
ADG (kg)	0.67 ± 0.04	0.74 ± 0.03	0.204
Backfat deposition (mm)	2.36 ± 0.28	3.58 ± 0.57	0.179

Values are means and standard error of the means (*n* = 7). Estrus (day), from the experiment beginning day to estrus day. Abbreviations: ADG, average daily gain; CONT, the basal diet; YETG, the basal diet + 0.25 g/kg yucca extract.

**Table 3:** Effects of YE on apparent digestibility of nutrients of gilts (%).

Items	CONT	YE	*p*-Values
GE	83.87 ± 0.91 ^b^	85.42 ± 0.89 ^a^	0.035
DM	84.13 ± 0.57 ^b^	85.65 ± 0.65 ^a^	0.047
EE	79.88 ± 0.97 ^b^	81.19 ± 1.02 ^a^	0.045
CP	83.25 ± 0.43 ^b^	84.52 ± 0.55 ^a^	0.038

Values are means and standard error of the means (*n* = 7). Abbreviations: GE, gross energy; DM, dry matter, EE, ether extract; CP, crude protein; CONT, the basal diet; YE, the basal diet + 0.25 g/kg yucca extract. In the same row, the absence of identical letters signifies significant differences (*p* < 0.05).

**Table 4:** Effects of YE on serum antioxidant capacity of gilts.

Items	CONT	YE	*p*-Values
T-AOC (U/mL)	0.62 ± 0.03 ^b^	1.26 ± 0.09 ^a^	*p* < 0.001
SOD (U/mL)	0.38 ± 0.00	0.37 ± 0.01	0.517
GSH-Px (U/mL)	1.38 ± 0.02	1.37 ± 0.01	0.572
MDA (μmol/mL)	0.65 ± 0.02 ^a^	0.39 ± 0.03 ^b^	*p* < 0.001

Values are means and standard error of the means (*n* = 7). Abbreviations: T-AOC, total antioxidant capacity; SOD, superoxide dismutase; GSH-Px, glutathione peroxidase; MDA, malondialdehyde; CONT, the basal diet; YE, the basal diet + 0.25 g/kg yucca extract. In the same row, the absence of identical letters signifies significant differences (*p* < 0.05).

**Table 5:** The effects of yucca extract on fecal microbial α diversity index of gilts.

Items	CONT	YE	*p*-Values
Observed species	1214.86 ± 40.99	1167.57 ± 37.94	0.414
Shannon	6.91 ± 0.22 ^b^	7.60 ± 0.08 ^a^	0.018
Simpson	0.94 ± 0.01 ^b^	0.97 ± 0.01 ^a^	0.042
Chao 1	1224.23 ± 40.68	1180.01 ± 40.15	0.454

Abbreviations: CONT, the basal diet; YE, the basal diet + 0.25 g/kg yucca extract. In the same row, the absence of identical letters signifies significant differences (*p* < 0.05).

**Table 6:** Effects of YE on the relative abundance of fecal microbiota levels at the phylum level of gilts.

Items	CONT	YE	*p*-Values
Firmicutes	0.65 ± 0.04	0.64 ± 0.04	0.846
Bacteroidota	0.20 ± 0.03	0.26 ± 0.03	0.187
Spirochaetota	0.09 ± 0.02	0.06 ± 0.01	0.241
Proteobacteria	0.029 ± 0.003 ^a^	0.017 ± 0.002 ^b^	0.010
Actinobacteriota	0.011 ± 0.001 ^a^	0.005 ± 0.001 ^b^	0.010
Euryarchaeota	0.003 ± 0.001	0.007 ± 0.002	0.179
Fusobacteriota	0.002 ± 0.001	0.001 ± 0.003	0.223
Acidobacteriota	0.002 ± 0.004	0.002 ± 0.003	0.200

Abbreviations: CONT, the basal diet; YE, the basal diet + 0.25 g/kg yucca extract. In the same row, the absence of identical letters signifies significant differences (*p* < 0.05).

**Table 7:** Effects of YE on the relative abundance of fecal microbiota levels at the genus level of gilts.

Items	CONT	YE	*p*-Values
*Streptococcus*	0.22 ± 0.04 ^a^	0.11 ± 0.03 ^b^	0.025
*Treponema*	0.09 ± 0.02	0.06 ± 0.00	0.241
*Muribaculaceae*	0.05 ± 0.01 ^b^	0.10 ± 0.01 ^a^	0.017
*Clostridium_sensu_stricto_1*	0.07 ± 0.01	0.10 ± 0.01	0.180
*Clostridia_UCG-014*	0.02 ± 0.00	0.03 ± 0.01	0.565
*p-251-o5*	0.03 ± 0.01	0.02 ± 0.01	0.848
*Prevotella*	0.01 ± 0.00 ^b^	0.02 ± 0.00 ^a^	0.013
*RF39*	0.007 ± 0.00 ^b^	0.010 ± 0.00 ^a^	0.030
*Prevotellaceae_NK3B31_group*	0.01 ± 0.00	0.02 ± 0.00	0.482
*NK4A214_group*	0.02 ± 0.00 ^b^	0.04 ± 0.00 ^a^	0.002

Abbreviations: CONT, the basal diet; YE, the basal diet + 0.25 g/kg yucca extract. In the same row, the absence of identical letters signifies significant differences (*p* < 0.05).

## Data Availability

The data presented in this study are available in the article.
